# Antifungal features and properties of chitosan/sandalwood oil Pickering emulsion coating stabilized by appropriate cellulose nanofiber dosage for fresh fruit application

**DOI:** 10.1038/s41598-021-98074-w

**Published:** 2021-09-16

**Authors:** Ata Aditya Wardana, Arisa Koga, Fumina Tanaka, Fumihiko Tanaka

**Affiliations:** 1 Graduate School of Bioresource and Bioenvironmental Sciences, Kyushu University, 744, Motooka, Nishi-ku, Fukuoka-shi, Fukuoka 819-0395 Japan; 2 Food Technology Department, Faculty of Engineering, Bina Nusantara University, Jakarta, 11480 Indonesia; 3 Laboratory of Postharvest Science, Faculty of Agriculture, Kyushu University, 744, Motooka, Nishi-ku, Fukuoka-shi, Fukuoka 819-0395 Japan

**Keywords:** Microbiology, Nanoscience and technology

## Abstract

A novel composite edible coating film was developed from 0.8% chitosan (CS) and 0.5% sandalwood oil (SEO). Cellulose nanofibers (CNFs) were used as a stabilizer agent of oil-in-water Pickering emulsion. We found four typical groups of CNF level-dependent emulsion stabilization, including (1) unstable emulsion in the absence of CNFs; (2) unstable emulsion (0.006–0.21% CNFs); (3) stable emulsion (0.24–0.31% CNFs); and (4) regular emulsion with the addition of surfactant. Confocal laser scanning microscopy was performed to reveal the characteristics of droplet diameter and morphology. Antifungal tests against *Botrytis cinerea* and *Penicillium digitatum*, between emulsion coating stabilized with CNFs (CS-SEOpick) and CS or CS-SEO was tested. The effective concentration of CNFs (0.24%) may improve the performance of CS coating and maintain CS-SEO antifungal activity synergistically confirmed with a series of assays (in vitro, in vivo, and membrane integrity changes). The incorporation of CNFs contributed to improve the functional properties of CS and SEO-loaded CS including light transmission at UV and visible light wavelengths and tensile strength. Atomic force microscopy and scanning electron microscopy were employed to characterize the biocompatibility of each coating film formulation. Emulsion-CNF stabilized coating may have potential applications for active coating for fresh fruit commodities.

## Introduction

In recent years, due to growing environmental concerns, edible films and coatings have attracted interest in place of petroleum-based packaging. To achieve satisfactory characteristics, it is required that the selected biomaterials can form continuous network structures during the film-forming process. Among edible film-making materials (polysaccharides, proteins, and lipids), chitosan (CS) has been receiving increasing research attention due to its favorable properties including biocompatibility and antimicrobial action^1, 2^. Despite the benefits have been demonstrated by CS-based films and coatings, the use of pure CS had a relatively limited antifungal activity^3^. Efforts to overcome the CS limitations, particularly antimicrobial improvements are ongoing^4^.

Reports have documented improved methods in order to enhance CS performance including by: oil incorporation^5, 6^; crosslinking^7, 8^; and blending with gelatin, quinoa protein, tara gum, and zein^3, 9–11^. Incorporation of essential oil (EO) has gained considerable interest due to its efficacy in increasing the antimicrobial performance of CS-based films and coatings^12, 13^. Indonesian sandalwood essential oil (SEO), extracted from *Santalum album* originating from the Papua area contains potential active compounds, such as α- and β-santalol. Earlier reports revealed the antifungal potency of SEO against *Trichophyton mentagrophytes*^14^, *Microsporum canis*^15^, and *Trichophyton rubrum*^16^. Furthermore, SEO, which is commonly utilized as a food flavoring and adjuvant, is permitted for use in food applications by the United States Food and Drug Administration (FDA), Flavor and Extract Manufacturers Association (FEMA), and the Council of Europe (CoE)^17^.

The development of essential oil-loaded biopolymer still remains a challenge due to its hydrophobic nature and volatility which reduce the stability and biological activity. The oil droplet size and distribution along longitudinal and transverse sections led to a reduction in the distance as a consequence of water evaporation via flocculation and/or coalescence pathways^18^. To overcome these limitations, a proper emulsion technique is essential. Pickering emulsion offers a prospective method to enhance the stability of the emulsion system by utilizing solid particles instead of surfactants^5, 19, 20^. The solid particles play a role in preventing the collision and aggregation of emulsion droplets by accumulation at the oil–water interface. Furthermore, that stability mechanism resulted in tight packing of irreversibly adsorbed particles at the interface thus reducing the diffusion surface area of lipid droplets^21^. The use of polysaccharide-based emulsifiers such as nanocellulose has been gaining interest due to their prospective features including hydrophobicity, high adsorption capacity, biodegradability, and biocompatibility. An appealing aspect of nanocellulose is its anisotropic fiber structure, allowing for stabilization of the oil–water interface at very low loading levels^22, 23^.

In terms of preparation and application of nanocellulose-stabilized emulsions on fresh fruit commodities, only limited studies have been reported. Deng et al. (2018) found that the use of 0.1% cellulose nanocrystal, 3% oleic acid, and 2% CS coating significantly (*P* < 0.05) delayed ripening and reduced senescent scalding of ‘Bartlett’ pears compared with Semperfresh™ coating during 3 months of storage^6^. Jung and his coworkers (2020) investigated effect of Pickering emulsion coating of ′Bartlett’ pears coated with 1% oleic acid, 0.1% cellulose nanocrystal, and 2% CS was suggested for delaying ripening and superficial scalding of fruit during the long-term cold storage^24^. However, these studies provided no information on fruit disease inhibition offered by the emulsified coating, such as antifungal properties. In fact, fungi cause decay in a wide range of fruit commodities.

Unfortunately, regardless of cellulose having a positive effect on the emulsion stabilizer, fungi possess some capacity to degrade cellulose resulting in a chain of glucose units that can be used for energy. Fungi use extracellular enzymes, cellulases, to break down cellulose into smaller chains, including cellobiose or glucose allowing for uptake across cell walls and use for metabolism^25, 26^. Therefore, determining the appropriate concentration of cellulose nanofibers (CNFs) is necessary to produce the functional properties-improved coating film without reducing the antifungal feature from CS-SEO composite coating. The objectives of this work were to: (1) develop an emulsified coating film formulation based on CS and SEO using a Pickering emulsion approach with an appropriate level of CNF as a stabilizer, (2) study and compare the antifungal features against *Botrytis cinerea* and *Penicillium digitatum* and film properties of CS, CS-SEO, and CS-SEOpick.

## Result

### Materials characterization

**Figure 1:**
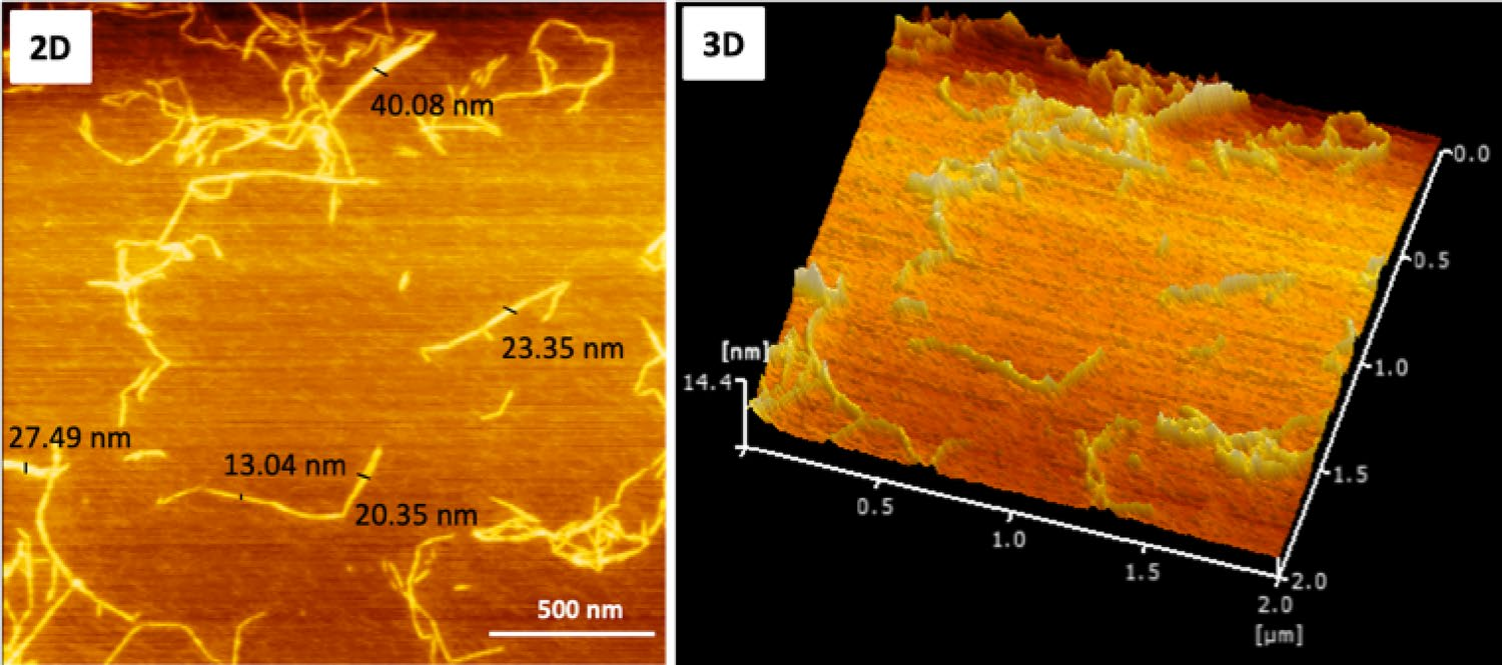
AFM images represent the morphology of two- and three-dimensional of CNF.

AFM images were taken to confirm the morphology and dimensions in the CNF suspension, as shown in Fig. 1. CNFs exhibited a typical rod-like structure, and agglomeration between individual cellulose fibrils occurred in some regions. CNFs possess a diameter in the nanometer scale and length in the micrometer scale and have both crystalline and amorphous sections^27^. In this study, the width of CNFs was approximately 13.04–40.08 nm, with an average of 24.86 ± 10.01 nm. Heterogenous sizes were shown for the length of individual CNFs. Although the length of CNFs cannot be estimated properly from AFM, it may be considered that the length of CNFs was in the micrometer scale.

### Emulsion stability and creaming behavior

**Figure 2:**
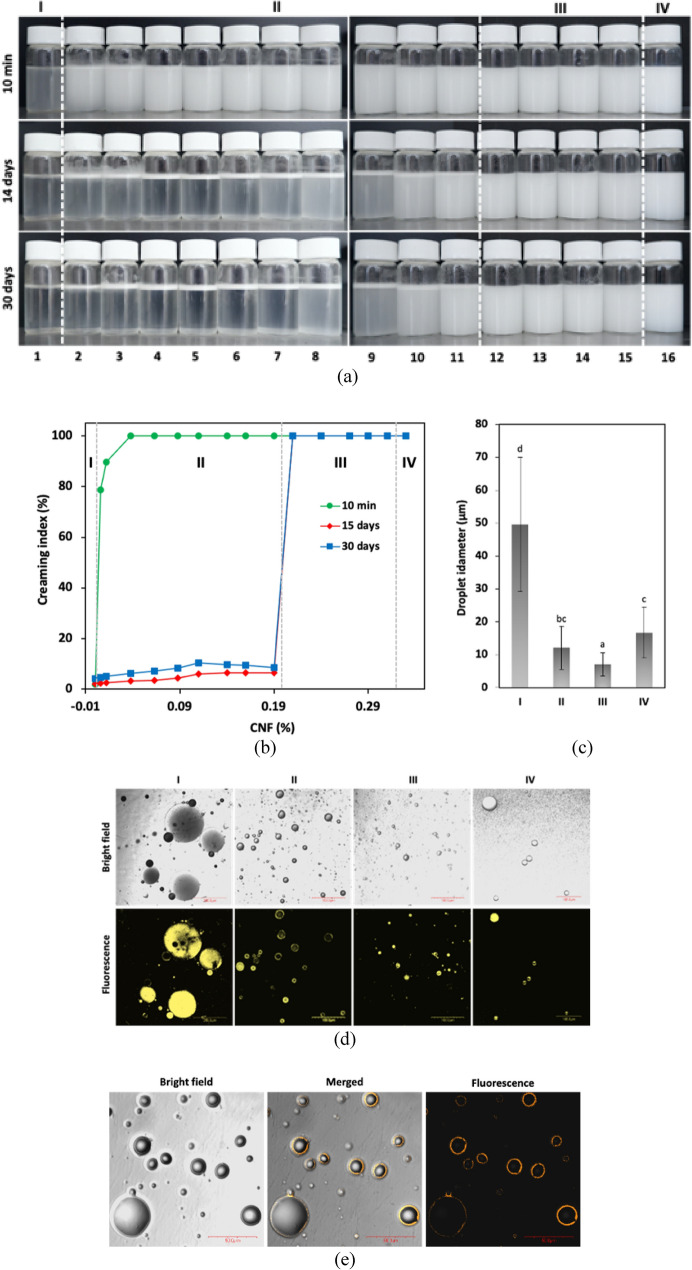
(**a**) Visual appearance of CNF-stabilized Pickering emulsions containing 0.5% SEO and CNF at various concentration of: 0, 0.006, 0.012, 0.038, 0.063, 0.088, 0.11, 0.14, 0.16, 0.19, 0.21, 0.24, 0.27, 0.29, 0.31% (from left to right, 1–15), and regular emulsion (16), and (**b**) their creaming index properties. (**c**) Droplet size distribution of emulsion, (**d**) confocal images of emulsion at day 7 after preparation, and (**e**) CNF-stabilized Pickering emulsion. The CNF was stained with acridine orange. Different letters indicate statistical significant differences at *P* < 0.05.

In this section, oil-in-water type emulsions were prepared by dispersing various levels of CNFs into aqueous solutions of CS-SEO. Figure 2a shows images of the emulsion samples at 10 min, and 14 and 30 days after preparation and with storage at ambient temperature. We divided these into several groups according to the typical stability of each emulsion, and we named these groups I, II, III, and IV. Group I exhibited a creaming index of the emulsion in the absence of CNFs (0% CNFs), which did not affect emulsion stability leading to the formation of a cream layer upon storage (negative control). Group II showed a creaming index of emulsion mixtures in the presence of CNFs (0.006–0.21%). In this group, we determined that when CNFs were added, the emulsification capacity was improved, indicating that CNFs played an essential role in stabilization of the emulsion, as shown after 10 min of storage (Fig. 2b). Even though the creaming index was gradually augmented with increasing CNF concentration, the emulsion was not stable and clearly separated in two different phases, as demonstrated at 14 days (Fig. 2b). Group III demonstrates the creaming index of emulsion mixtures in the presence 0.24–0.31% CNF, demonstrating the absence of creaming after the storage period because of the emulsion-stabilizing effect of CNFs in the emulsion system. The ability of emulsion stabilization of Group III was the same as Group IV, which formed a regular emulsion (positive control). We assumed that the concentration range of CNFs used in group III provided the optimum result in the context of the Pickering emulsion effect.

### Morphology and droplet size

The microstructure of emulsified coating samples was investigated using confocal laser scanning microscopy (CLSM) obtained from the top creaming layers of the emulsion system. Obviously, the emulsion droplets were distributed in the CLSM photograph, the droplets had a spherical shape with various particle sizes ranging from 7.02 to 49.63 μm (Fig. 2c). Figure 2d shows that with increasing contents of CNFs, the droplets size gradually decreased compared with both negative and positive control emulsions. Notably, the oil droplet size in group III was more homogeneously distributed and significantly (*P* < 0.05) lower that regular emulsion.

### The morphological structure of Pickering emulsion stabilized by CNFs

CLSM was used to further understand the deeper morphological structure of Pickering emulsions and the location of CNFs in the emulsion system. As seen in Fig. 2e in bright field mode, the distribution of spherical droplets of SEO was clearly captured. When monitored using merged mode, orange fluorescence from acridine orange stained-CNFs lay in the surface of the dispersed phase. In the fluorescence image, orange circles around spherical droplets were more clearly observed at SEO–water boundaries.

### Effect of coating treatment on mycelial growth inhibition

**Figure 3:**
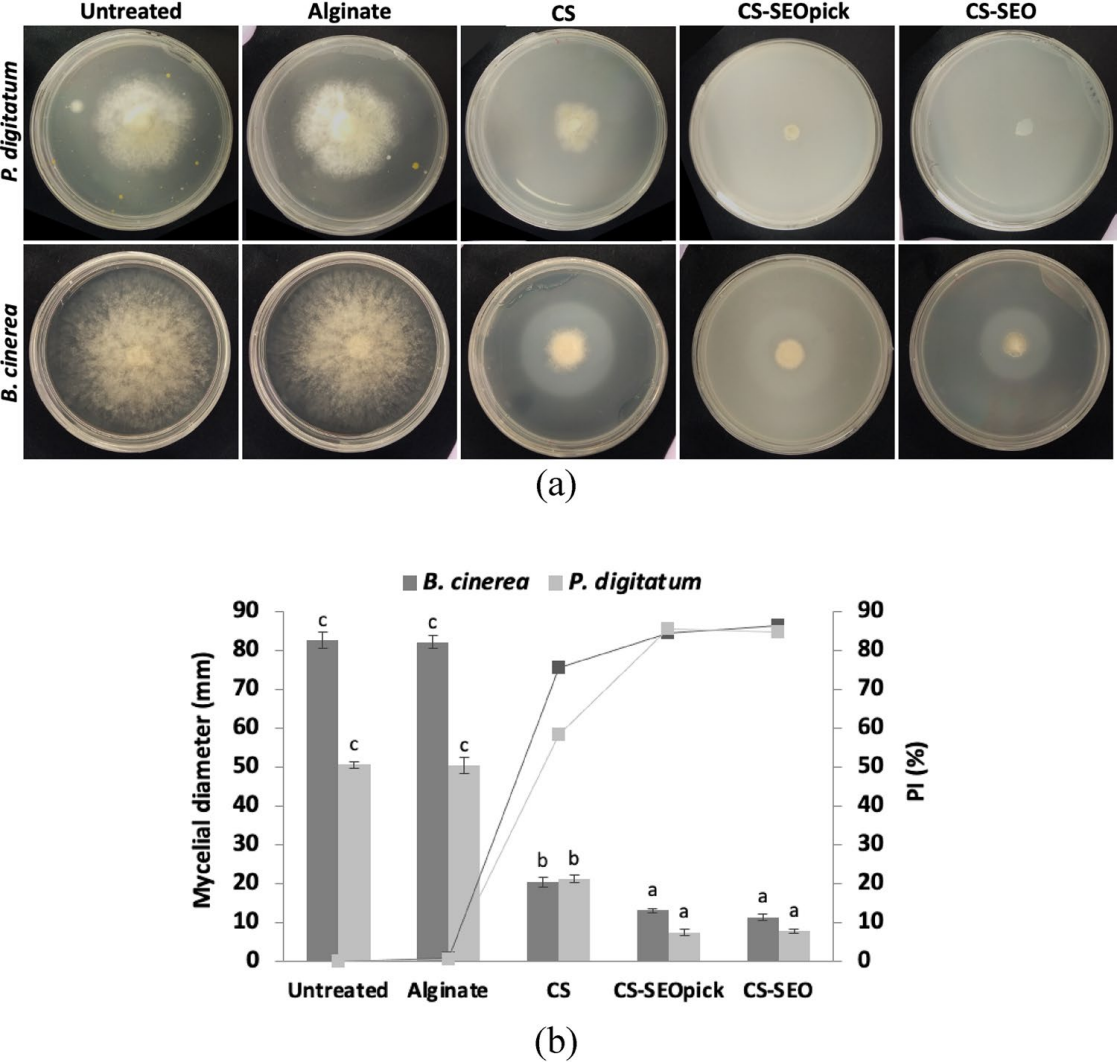
Influence of coating treatment on mycelial growth inhibition of *P. digitatum* and *B. cinerea* visually (**a**) and statistically (**b**) in the day 5. Different letters indicate statistically significant differences among different treatments at *P* < 0.05. *CS* chitosan, *CS-SEOpick* chitosan–sandalwood oil Pickering emulsion, *CS-SEO* chitosan–sandalwood oil regular emulsion treated spores. Alginate represents the common commercial edible coating.

The mycelial growth and extension of *P. digitatum* and *B. cinerea* in solid medium were controlled in response to the coating treatment, as demonstrated in Fig. 3. The results indicated that coating restricted the spread of mycelia significantly (*P* < 0.05) compared with the untreated and alginate representing the common commercial edible coating. Improvement occurred when CS was combined with SEO, mycelium extension decreased significantly (*P* < 0.05) at the end of incubation. There was no significant difference (*P* > 0.05) when the CNFs was loaded.

### Effect of coating treatment on fungal growth

**Figure 4:**
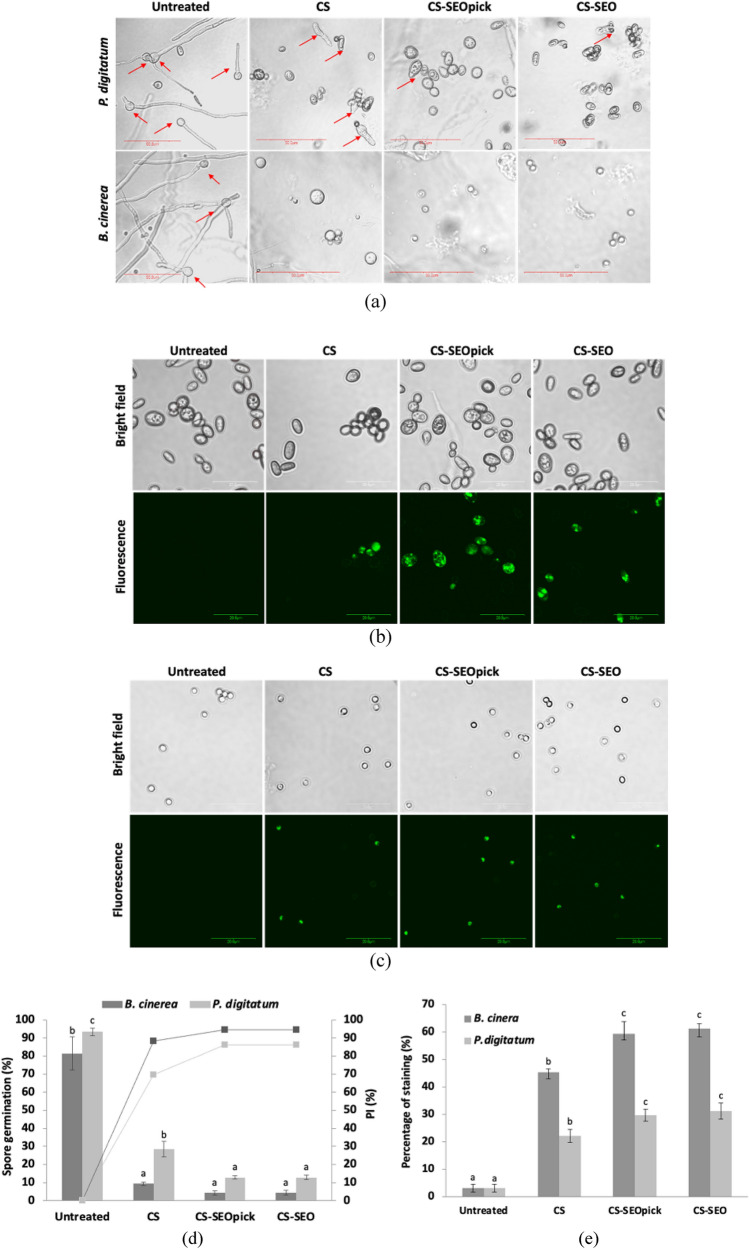
Effect of coating treatment for the decrease in spore survival of *P. digitatum* and *B. cinerea* visually (**a**) and statistically (**d**). Detection of membrane integrity visually of *P. digitatum* (**b**), *B. cinerea* (**c**), and statistically (**e**). Different letters indicate statistically significant differences among different treatments at *P* < 0.05. *CS* chitosan, *CS-SEOpick* chitosan–sandalwood oil Pickering emulsion, *CS-SEO* chitosan–sandalwood oil regular emulsion treated spores.

Visually, *B. cinerea* spores were more susceptible in the presence of coating solution than those of *P. digitatum* (Fig. 4a). All spores germinated easily when the coating treatment was absent. No completely germinated spores were observed among the treated spores of *B. cinerea*, even though some CS-treated spores were seen starting to germinate, as indicated by swelling of the spores. A spore was categorized as germinated when the longest germ tube length was equal to or greater than the largest dimension of the swollen spore^28^. Unlike spores of *B. cinerea*, some spores of *P. digitatum* were able to germinate, as indicated with red arrows, and the number was reduced with the incorporation of SEO into CS, indicating higher inhibition. Statistically, comparing with untreated spores, the inhibition of spore germination of *B. cinerea* treated with CS increased dramatically by 88.32% (Fig. 4d). No difference in spore survival inhibition was seen when CS was augmented with SEO and CNFs. In the case of *P. digitatum*, CS-coating treatment also effectively reduced percentage of spore germination from 93.5% (untreated) to 28.41% (CS treated), with percentage inhibition (*PI*) a value 69.55%. Furthermore, SEO-containing CS showed synergistically improved antifungal performance that inhibited the growth of *P. digitatum* spores with 12.87% germination and a *PI* value 86.24%. There was no significant difference (*P* > 0.05) when the Pickering emulsion agent, CNFs, was added.

### Effects of coating on fungal membrane permeability

Propidium iodide can penetrate dead cells with damaged plasma membranes, resulting fluorescent staining^29, 30^. As a result, untreated spores were not stained, indicating that they were healthy spores (Fig. 4b,c), whereas coating-treated spores were readily stained, and a significant difference demonstrated (*P* < 0.05) compared with the control (untreated). This method is applicable for all type of spores (*P. digitatum* and *B. cinerea*). A significant increase (*P* < 0.05) in the percentage of stained cells with green fluorescence occurred when they were treated with CS-SEO or CS-SEOpick coatings (Fig. 4e) in comparison with CS treatments, indicating a better antifungal action.

### Antifungal performance of coating on tangerine and apple fruit in vivo

**Figure 5:**
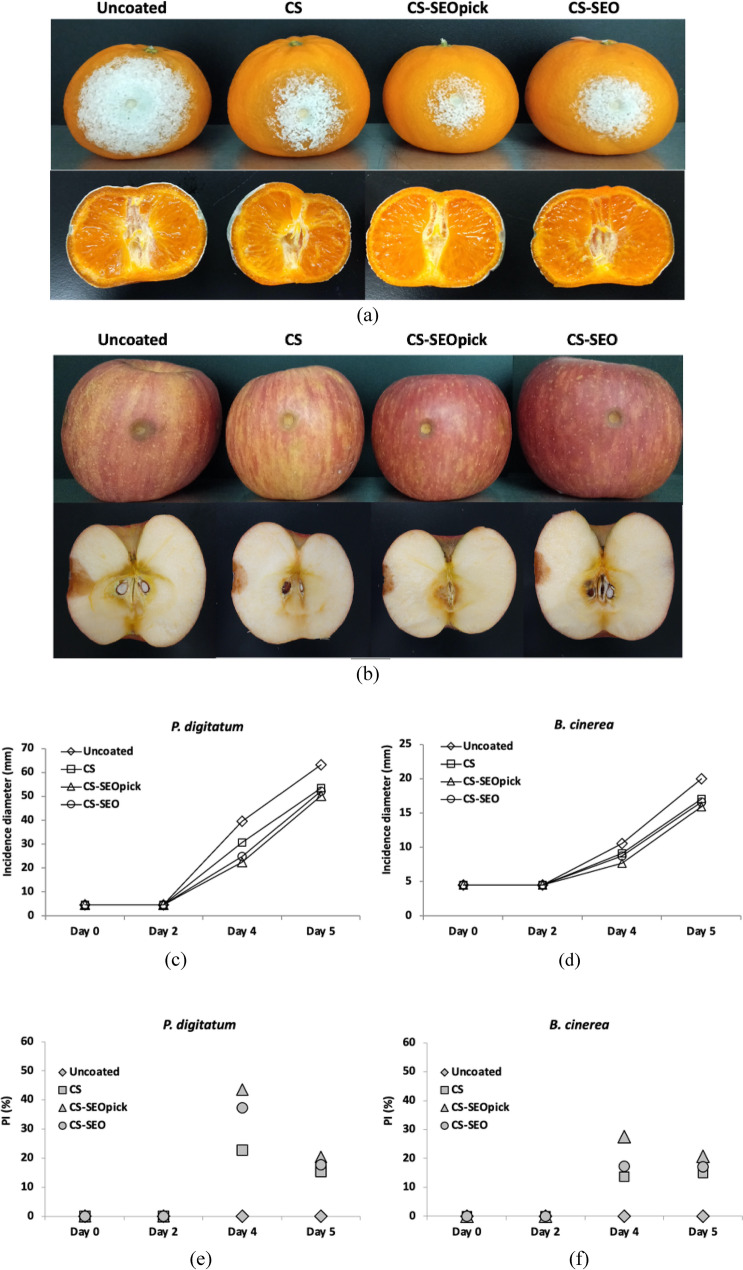
Efficacy of various coating treatment against *P. digitatum* and *B. cinerea* disease severity. (**a**, **b**) Visual appearance of representative sample and (**c**, **d**) statistical analysis during storage. *PI* percentage inhibition, *CS* chitosan, *CS-SEOpick* chitosan–sandalwood oil Pickering emulsion, *CS-SEO* chitosan–sandalwood oil regular emulsion coated fruits.

During storage for 5 days, all fruits exhibited similar trends of decay, in which is mold symptoms and growth increased (Fig. 5a,b). Until day 2 of storage, no mycelial expansion appeared on uncoated (control) and coated fruits, indicating a lag phase of mold. On day 4, lesion diameter increased drastically on the surface of fruit, ranging from 22.26 to 39.49 mm and from 7.63 to 10.55 mm on tangerine and apple, respectively (Fig. 5c,d). The hyphal expansion of *P. digitatum* was delayed significantly (*P* < 0.05) by CS coating treatment with a *PI* value 27.71% higher than the control. The inhibition effect of CS also occurred for *B. cinerea* by 13.68% compared with untreated fruit. Furthermore, SEO-containing CS showed synergistically improved antifungal activity in vivo with *PI* values of 37.21% and 43.61% for CS-SEO- and CS-SEOpick-coated tangerines. The result was slightly different for *B. cinerea*-infected fruit, with no improvement in the inhibition of lesion extension by coating treatment between CS and CS-SEO except after the inclusion of Pickering emulsion with CNFs. The trend in mycelial expansion diameter steadily increased until the final day of storage (day 5). Surprisingly, the *PI* values for *P. digitatum* and *B. cinerea* growth on day 5 were lower than on day 4 (Fig. 5e,f). Through observation of cross-sections of infected tangerines, the penetration growth of internal fungal decay showed similar appearances for all samples. Decay with *B. cinerea* showed higher penetration in control fruit than CS, CS-SEO-coated apples, and the growth of internal decay of CS-SEOpick-coated fruit was the lowest.

### Color parameters

As presented in Table 1, the a* value was negative for all films and decreased significantly (*P* < 0.05) with the addition of CNFs. The b* value (blue–yellow color), which varied from 6.94 to 15.22, indicating a slight yellowish color to the films, increased with the addition of SEO and CNFs (*P* < 0.05). The L* value decreased slightly due to the incorporation of SEO and CNFs into the CS film (*P* < 0.05). It was documented that EO or cellulose-enriched edible film may enhance the brightness visually, indicated by lower L* value and greater ∆E* value^31, 32^, in which our results were consistent with this phenomenon.

**Table 1:** Characteristics of developed films.

Characteristic	CS	CS-SEOpick	CS-SEO
L*	98.2 ± 0.32^c^	96.52 ± 0.36^a^	97.18 ± 0.22^b^
a*	− 0.12 ± 0.18^b^	− 0.7 ± 0.16^a^	− 0.14 ± 0.05^b^
b*	6.94 ± 0.72^a^	15.22 ± 0.84^c^	9.8 ± 1.29^b^
ΔE*	3.02 ± 0.65^a^	11.48 ± 0.75^c^	6.03 ± 1.12^b^
Tensile strength (MPa)	9.02 ± 0.73^a^	12.41 ± 1.74^b^	10.18 ± 1.29^a^
Elongation (%)	33.02 ± 4.47^a^	31.42 ± 2.19^a^	39.01 ± 4.18^b^
*Ra*	4.07 ± 2.20	5.44 ± 1.08	4.26 ± 1.99
*Rq*	4.47 ± 2.77	6.86 ± 1.21	5.47 ± 2.61

*CS* chitosan, *CS-SEOpick* chitosan–sandalwood oil Pickering emulsion, *CS-SEO* chitosan–sandalwood oil regular emulsion films.

Different letters indicate statistically significant differences at *P* < 0.05.

### Light transmittance, and opacity

**Figure 6:**
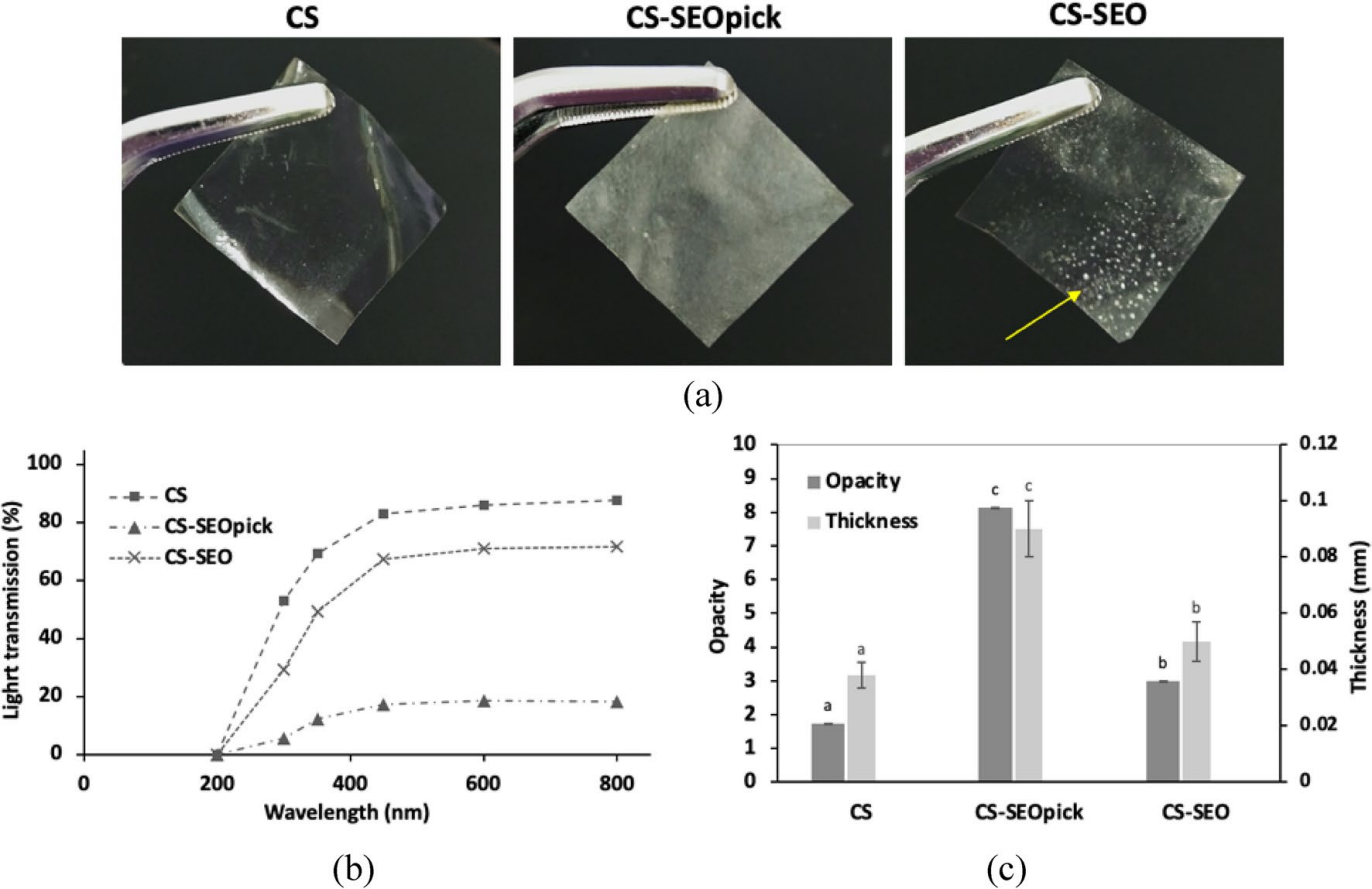
Visual photographs of coating film appearance (**a**) and its optical properties; light transmittance (**b**) and opacity and thickness for thin coating film (**c**). Different letters indicate statistically significant differences at *P* < 0.05. *CS* chitosan, *CS-SEOpick* chitosan–sandalwood oil Pickering emulsion, *CS-SEO* chitosan–sandalwood oil regular emulsion films.

The CS film showed the highest clarity with transmittance between 82.84 and 87.59% in the visible wavelengths. Compared to CS alone, CS-SEO had a 18.22 ± 0.61% lower transmittance at all visible light wavelengths; therefore, significantly higher opacity was exhibited (Fig. 6b,c). Moreover, sweating-out of SEO, indicated with a yellow arrow, from the inside to the surface of the CS-SEO film was seen clearly with the naked eye (Fig. 6a). The highest reduction in light transmission was demonstrated with CS-SEOpick with lower transmittance values of 78.91 ± 0.34% and 74.21 ± 0.31% compared with CS and CS-SEO, respectively. Consequently, the opacity value was dramatically decreased. The average thickness of CS was 0.038 ± 0.004 mm, and the addition of SEO increased this value to a significant extent (*P* < 0.05). CS-SEOpick showed the highest thickness at an average value of 0.09 ± 0.01 mm.

### Mechanical properties

The tensile strength of film was enhanced significantly (*P* < 0.05) from 9.02 ± 0.73 MPa and 10.17 ± 1.29 MPa for CS and CS-SEO films up to 12.41 ± 1.74 MPa (Table 1). The elongation of CS films (33 ± 0.73%), indicating the flexibility of the films, was not altered by the addition of CNFs (31.4 ± 1.74%). However, significant (*P* < 0.05) enhancement of the elongation value was found when 0.5% SEO was added.

### Surface morphology

**Figure 7:**
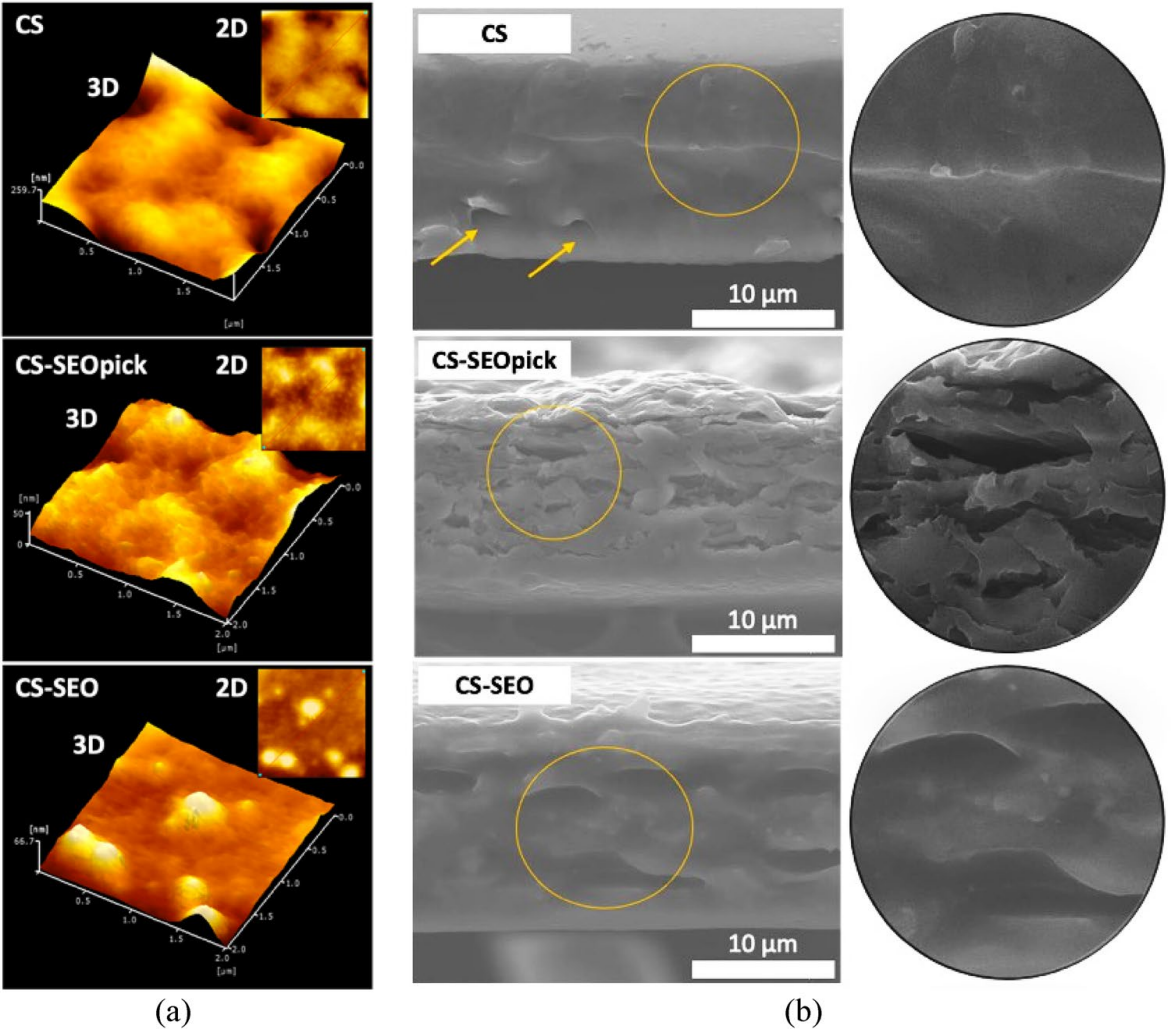
(**a**) AFM topographic image and (**b**) SEM longitudinal cross section image. *CS* chitosan, *CS-SEOpick* chitosan–sandalwood oil Pickering emulsion, *CS-SEO* chitosan–sandalwood oil regular emulsion films.

As seen in Fig. 7a, although no significant differences were demonstrated (*P* > 0.05), there was a tendency from an increasingly smooth surface in CS film with average Ra and Rq values of 4.07 ± 2.20 and 4.47 ± 2.77 nm, respectively. The surface was rougher, Ra = 4.26 ± 1.99 nm and Rq = 5.47 ± 2.61 nm, when SEO droplets were inserted into the CS matrix. A less smooth surface, Ra = 5.54 ± 1.08 nm and Rq = 6.86 ± 1.21 nm, appeared with the addition CNFs as a Pickering emulsion agent.

### Scanning electron microscopy

CS film obtained without CNF or SEO displayed a compact, continuous, and homogenous microstructure, without cracks, and almost no obvious separation was detected, showed in Fig. 7b. There were two slight cavity-like structures found (indicated by yellow arrows), which were probably caused by the sample preparation procedure. The incorporation of SEO resulted in an amorphous structure, with vacuoles and pores distributed along the cross-section surface of CS-SEOpick and CS-SEO. Notably, the dispersion of SEO developed with Pickering emulsion caused a decrease in the discontinuities and droplet size and an increase the droplet distribution in the cross-sections of films, as indicated with a yellow circle.

## Discussion

In this study, a novel coating film was fabricated successfully, with the main objective to develop a stable emulsified coating to maintain the quality of fresh fruit by preventing fungal decay. We performed preliminary tests for emulsion stability and creaming behavior in selecting the optimum concentration of CNFs for further development of the coating film. Considering the above results, the use of 0.24% CNFs (from group III) was preferable from the stabilization improvement and economic point of view. Previous work investigated the stability mechanism of cellulose and found that cellulose adsorbed at the oil–water interface induced attraction and aggregation phenomena under the interfacial disturbance, as confirmed with the interfacial rheological properties^33^. An improved stability mechanism was also proposed, which was related to the coverage effect from CNF–carboxymethyl CS complexes, allowing irreversible adsorption on a beeswax–water interface preventing coalescence or creaming as a result of the dense three-dimensional network^34^.

Morphology monitoring using CLSM revealed that orange fluorescence indicating acridine orange stained-CNFs lay at the surface of the dispersed phase, thereby stabilizing the oil droplets. This use of CLSM to confirm the template of the Pickering emulsion agent was also reported for zein particles stained with Nile blue A^5^, CNFs and nanocrystals stained with calcofluor white^35^, and nanocellulose stained with acridine orange^36^. Furthermore, CNFs affected the oil droplet size, indicating that CNFs had an important role in preventing the collision and aggregation of emulsion droplets by their accumulation at the oil–water interface. Again, the above results suggested that the incorporation of 0.24% CNFs may be an ideal stabilizer candidate to maintain demulsification of the coating film solution.

*B. cinerea* and *P. digitatum* are regarded as the most important fresh fruit fungal pathogens; therefore, in this study the inhibition of fungal growth was tested. We can assume that the use of CS alone without the addition of EO was sufficient to suppress *B. cinerea* spore growth (Fig. 4). However, it was slightly different compared to the mycelial growth inhibition. The CS-microbial penetration was probably lower compared with the liquid phase, resulting a lower efficacy (Fig. 3). Proposed mechanisms have been documented to explain the antifungal performance of CS, including: electrostatic interactions between positively charged CS molecules and negatively charged fungal cell walls resulting in ionic imbalance^37, 38^; intracellular interactivity of CS and DNA allowing the disruption of mRNA and protein synthesis^39, 40^; and the chelating ability of CS for metals that are essential as microbial nutrients^41, 42^. In the case of *P. digitatum*, SEO-incorporated CS demonstrated synergistically improved antifungal action. Similar findings have been reported when lemongrass or clove EO was entrapped in CS^43^. The active components, α- and β-santalol, are believed to be potent antifungal agents contained in SEO. These sesquiterpenoid compounds may disturb fungal cell wall synthesis and have been confirmed by the occurrence of abnormal swelling and curling of terminal hyphae of test fungi^16^. Notably, no difference was found when a Pickering emulsion agent, CNFs, was added. Naturally, cellulose can be used by fungi for energy. Chains of glucose units obtained from cellulose degradation using extracellular cellulases is taken up across the fungal cell wall and metabolized^25, 26, 44^. There are two proposed possibilities associated with these phenomena, (1) the use of CNFs in this study was at appropriate concentration as a Pickering agent without reducing the antifungal features of the CS-SEO composite coating, and (2) CNFs may inhibit the loss of SEO by protecting the oil droplets from environmental exposure. A propidium iodide staining test was used to better confirm the effects of coating treatment for the loss of membrane integrity in fungal spores. Because propidium iodide is membrane impermeable, the results (Fig. 4b,c) implied that membrane integrity was interfered with by coating treatment, which led to metabolic disruption and the death of fungi.

To confirm the antifungal efficacy of the coating treatment, in vivo tests on tangerine and apple fruit that had been artificially contaminated with *P. digitatum* and *B. cinerea*, respectively, were carried out. *B. cinerea* is a ubiquitous microorganism that is the main cause of postharvest disease, thereby causing considerable losses in harvested fruit. Green mold *P. digitatum* is well known as a pathogen inflicting major postharvest disease on citrus fruits and for being resistant to different fungicides^45^. The result reflected a coherent efficacy profile from the results of spore germination (Fig. 4). Again, there was clear evidence of the appropriate level of CNFs as a Pickering agent and the improvement of functional properties in this study, suggesting that the components have an effective antifungal action. There was a surprising finding that the PI values at day 5 were lower than at day 4. This might be ascribed to the availability of intrinsic carbon in plant hosts^46^. On the final storage day, a higher number of carbon source may be available in coated fruits, whereas molds have exceeded the adaptation phase in the host environment. This lesion incidence also has implications for fruit internal decay.

In practice, film coatings are applied either directly onto the surface of food forming a thin layer film or as a stand-alone wrapping material; therefore, the color properties of the thin film (L*, a*, b*) and ΔE* were characterized. Overall, the value of (L*, a*, b*) and ΔE* were altered by the addition of SEO and CNF into CS films. Based on the results for ΔE*, people were able to easily perceive a visual color difference with the prepared coating films. A literature noted that at the limit of ΔE* = 2, the human eye is not able to distinguish the color of each coating film with the naked eye^47^.

The light transmission parameter reflects the barrier ability of the developed film against UV and visible light. It is one of the essential features associated with the potency of a coating film is its ability to inhibit the oxidation of lipids, pigments, proteins, or vitamins in packed foods^48^. The incorporation of SEO and/or CNFs decreased CS transmittance throughout the visible light range, consequently higher opacity was exhibited. Earlier study explained that higher opacity was due to the presence of oil droplets in the film matrix that scattered the transmitted light^32^. Moreover, sweating-out (exudation) of SEO from the biopolymer matrix film onto the surface may also contribute to the decreasing transparency value of the CS-SEO composite film. The intermolecular interactions between SEO and water or biopolymer matrix mediated by Tween 80 were diminished as the water content decreased during film formation (drying process). Thereby, the dispersed phase (oil) was not immobilized, leading to migration to the surface of the film. In the case of CNF-stabilized film, it is likely that CNF addition affected the internal and surface structure, and efficiently blocked the visible spectrum. The enhancement of the opacity of edible films was positively correlated with the addition of CNF which lead to light scattering^49^. All coating films showed lower light transmission values with UV light than in the visible light spectrum, with a similar trend except at 200 nm. From this result, it can be assumed that the CS-SEOpick film had higher barrier properties for UV and visible light. Moreover, the thickness of a film is a predominant factor affecting the optical characteristics (Fig. 6c). The changes in thickness were probably due to an increase in the total solid content of the films.

The use of CNF as a Pickering emulsion agent increased the tensile strength of the films. Not only due to the geometry and rigidity of the nano-filler but also the enhancement in tensile strength was also associated with the formation of a stiff continuous network of CNFs linked through hydrogen bonding^50^. In addition, nano-sized cellulose can readily form hydrogen bonds with the surrounding molecules, leading to strong interactions even at low concentration as a result of the large aspect ratio and ability to form interconnected network structures^51, 52^. However, another study investigated whether the reinforcing efficacy of CNFs declined when crystalline nanocellulose was incorporated into alginate^53^ and pectin^53^ at higher concentration (> 5% w/w on solid polymer) due to the agglomeration and non-uniform dispersion of the filler. The level of CNF used in this study was assumed to be in the optimum concentration range for the filler in improving the tensile strength of the coating film. There were no significant changes of CS elongation due to the addition of CNFs. In this investigation, the emulsified films had higher elongation, and this was in agreement with other investigations^54, 55^. This behavior may be attributed with a sufficient level essential oil resulting in a synergistic impact of the plasticizer and SEO. Essential oil can perform as a plasticizer agent allowing greater mobility and flexibility in the polymer chain^54^.

Cross-section analysis was performed to observe the microstructural arrangement of the films and to confirm the distribution of SEO droplets and CNF entrapped in the biopolymer. The discontinuities demonstrated in SEO-loaded films may be associated with SEO microdrop. Similar findings in the presence of the oil micro-droplets of the hazelnut meal protein matrix, observed with the cross-section of a film^56^. Furthermore, the SEO droplets obtained in this study showed a shrinkage-like shape of an oval structure (Fig. 7b). The existence of these types of pore droplets might correspond to a consequence of the drying process and the density of the coating film matrix^56, 57^. A decrease in discontinuities and droplet size and an increase in droplet distribution was found in CS-SEOpick film. This phenomenon possibly corresponded to the occurrence of flocculation and coagulation from the dispersed phase in CS-SEO. The emulsion system in CS-SEOpick was believed to have a higher physical stability compared with the regular emulsion mediated by surfactant (CS-SEO). This suggested that the presence of CNFs may have fully covered the oil droplets allowing the prevention of oil droplet coalescence. The lower size of cellulose was noted to be more easily adsorbed onto the oil–water interface, hence facilitating the higher stability of oil in water emulsion formation^21^. These stabilization mechanisms not only minimized the evaporation of SEO during the drying process but also protected against the oxidation of SEO.

AFM analysis is a powerful method to analyze the occurrence of slight changes in the surface of films as a consequence of filler material incorporation both qualitatively and quantitatively^58^. Reduced smoothness of the CS coating surface after the addition of SEO might be ascribed to lipid aggregation and/or creaming phenomena. Furthermore, these phenomena were exacerbated by an evaporation step during film formation, thereby the level of irregularities on the films' surfaces increased^59^. This was confirmed visually by 2D and 3D topography image and line profiles which oil aggregates are exist forming several spherical uplands structure. A previous study also found an increase in terms of film roughness because of the incorporation of oregano oil into gelatin-chitosan blend film^60^. Furthermore, the roughness value also increased with the incorporation of CNFs. An earlier study proposed that nanocellulose might be aggregated, implying the higher roughness of the film through the formation of uplands in the surface^61^. This trend was in good agreement with another report which observed the agglomeration of cellulose nanocrystals in the pectin matrix^62^. However, as seen in Fig. 7a, CNFs were dispersed uniformly on the surface of the CS-SEO composite films assuming a good matrix-CNF interaction and confirming the improvement in mechanical properties (tensile strength). A slight increase and insignificant difference in roughness implies the ideal concentration of CNFs used in this study without prejudice from its main function as an emulsifier.

In conclusion, novel composite coating formulations using 0.8% CS/0.5% SEO/0.24% CNF were developed. The incorporation of 0.24% CNF as a stabilizer agent into chitosan/sandalwood oil Pickering emulsion coating was found to improve the performance of antifungal activity synergistically confirmed with in vitro and in vivo assays. This work also revealed that the addition of CNF had potential to improve the functional properties such as light transmission at UV and visible light wavelengths and tensile strength. Additional investigation should be performed, particularly for physico-chemical characteristics and sensory acceptability, to confirm the potential of this novel active coating in maintaining the quality of fresh fruit commodities.

## Methods

### Materials

Chitosan (CS), Tween 80, and glacial acetic acid were obtained from FUJIFILM Wako Pure Chemical Corporation, Japan. CNF powder, made from wood-derived fiber, was obtained from Nippon Paper Industries Co., Ltd, Japan. That CNF was chemically treated by 2,2,6,6-tetramethylpiperidine-1-oxyl (TEMPO) catalytic oxidation method. Papua sandalwood essential oil (SEO) was obtained from Aromindo CV, Indonesia, with the two main contents, α-santalol (19.36%) and β-santalol (16.48%), identified using a gas chromatography (GC)-mass spectrometer (Shimadzu QP 2010 Plus, Japan) equipped with capillary column of 0.25 mm i.d., length 30 m, film thickness 0.25 μm (DB-5MS column, J. & W. Scientific, USA).

### Preparation of SEO Pickering emulsions and coating

The stabilized Pickering emulsion agent stock (CNF_s_) was prepared by dispersing CNFs at various concentrations into distilled water using high speed stirring at 15,000 rpm for 1 min with a high-speed homogenizer (T 25 digital ULTRA-TURRAX^®^—IKA, Germany). At the same time, the SEO stock (SEO_s_) solution was prepared using same procedure. Then, CNF_s_, SEO_s_, and distilled water were homogenized at 15,000 rpm for 5 min with a high-speed homogenizer to produce emulsion containing 0.5% SEO and various concentration of CNFs (0, 0.006%, 0.012%, 0.038%, 0.063%, 0.088%, 0.11%, 0.14%, 0.16%, 0.19%, 0.21%, 0.24%, 0.27%, 0.29%, and 0.31%). The emulsion containing 0.25% surfactant Tween 80 was used as a positive control representing a regular emulsion.

The stabilized Pickering emulsion coating film (CS-SEOpick) was prepared by mixing SEO_s_ (0.5%), and CNFs (0.24%) at 15,000 rpm for 3 min to form solid particles-stabilized SEO at the oil/water interface. The stabilized SEO droplets were inserted into the main matrix, CS solution, using a homogenizer at 15,000 rpm for 2 min, then degassed in a vacuum oven (ADP300, Yamato Scientific Co, Ltd, Japan). CS solution was prepared by gelatinizing CS powder in glacial acetic acid solution (1% v/v) followed by adding 2 N NaOH solution until pH 6. For some analyses, the thin films were produced using the casting method on a silicon molds plate (8 × 8 cm), dried at 40 °C for 15 h and peeled.

### Atomic force microscopy

The CNF morphology and the coating roughness were measured using AFM (Hitachi 5200S, Japan) equipped with the Nano Navi Application program, operating in dynamic force mode. Respectively, 3 µL of CNF solution (0.01% CNF in distilled water) and 10 µL of coating solution were dripped onto freshly precleaned mica, and dried and stored in a silica gel-containing desiccator for 24 h before imaging. Samples were scanned in noncontact mode using a sharpened cantilever type SI-DF20, scanning frequency 0.7–0.84 Hz, and scanning area 2 μm × 2 μm. Roughness characteristics including root mean square deviation from the mean (*Rq*) and arithmetical mean deviation from the mean (*Ra*) were examined using ten replicates and calculated as follows:

1 $$Rq = \sqrt {\frac{1}{n} \mathop \sum \limits_{i = 1}^{n} Zi^{2} }$$

2 $$Ra=\frac{1}{n} \sum_{i=1}^{n}|Zi|$$

where *Zi* was the height deviation of *i*-th and *n* was the total of data points.

### Emulsion stability and creaming behavior

The emulsion stability of the coating solutions were determined visually referring to the photographed images taken after 10 min, 14 and 30 days of storage in room temperature. The percentage of creaming index was calculated following the method from Wand and Hauzei (2016).

3 $$CI =\frac{HS}{HE}\times 100\%$$

where *HS* is the height of the emulsion layer, *HE* is the total height of the coating solution.

### The microstructure and droplet size of Pickering emulsion layer

The oil droplet and layer of the Pickering emulsion were observed using CLSM with 20× objective lens (Olympus IX71, Japan). The top layer of the emulsion samples was stained with Nile red (FUJIFILM Wako Pure Chemical Corporation, Japan) solution (1 mg/mL in ethanol) to indicate the oil phase. CNFs were dyed with 0.1% acridine orange (FUJIFILM Wako Pure Chemical Corporation, Japan) before emulsion preparation. Then, 6 µL of each dyed sample was dropped gently onto a glass slide (Toshinriko, Japan) and covered with a thin coverslip (thickness ≈ 170 µm) (Matsunami, Japan). The excitation/emission spectrum for Nile red and acridine orange were 365 nm and 435 nm, respectively. The mean of diameter of oil droplet size in the Pickering emulsions was measured with the aid of ImageJ software from 30 of individual droplets.

### Antifungal assays

The agar dilution technique was used to test in vitro antifungal activities. Solid media was firstly prepared by mixing each coating solution and potato dextrose agar at a ratio of 1:1 and poured into sterilized petri dishes. After solidification, the 7-mm-agar disc with *P. digitatum* and *B. cinerea* mycelium, were placed on the center of each petri dish. Then they were incubated at 25 °C and the diameter of the colony zone was measured after 5 days.

The antifungal action was also determined using a spore germination test. The spore solution 5 × 10^6^ spores/mL of *P. digitatum* or *B. cinerea* from the National Institute of Technology and Evaluation, Biological Resource Center, Tokyo, Japan was mixed with distilled water (untreated sample) or coating solution, and potato dextrose broth (Difco™, USA) with a ratio 1:1:1. Fungal spores will break dormancy and begin to germinate once exposed to favorable conditions. The germinated spore was evaluated under CLSM with a 50× objective lens after shaking-incubated for 24 h, 100 rpm at room temperature.

4 $$Spore\,\, germination\, \% =\frac{Germinated\,\, spores}{Total \,\,spores }\times 100\%$$

where the total spores of each observation were around 200–250 spores. The result was analyzed from triplicate observations.

The in vivo test was determined by observing the lesion diameter on tangerine and apple fruits. Fruits used in the protocol comply with relevant institutional, national, and international guidelines and legislation. Fresh fruits, purchased from local supermarket in Fukuoka, Japan, were washed into 1% sodium hypochlorite solution for 10 min followed by air drying. Each fruit was wounded using a sterile corkborer (4 mm diameter and 2 mm deep) in the central region. About 10 µL of the spore solution was deposited in each wound. After air drying for 5 h, the whole surfaces of fruits were sprayed evenly on each side (± 2 mL/fruit) with distilled water (control) or coating solution, which was allowed to dry. Untreated and coated fruits were then kept at 25 °C and 90% RH for 5 days. The percentage inhibition (PI) was also calculated for all antifungal assays.

5 $$PI \% =\frac{A-B}{A }\times 100\%$$

where *A* is the maximum of the germinated spore (untreated sample) or lesion diameters and *B* is the germinated spore or lesion diameter obtained from each coating treatment. The result was analyzed from triplicate measurements.

### Membrane integrity

Membrane integrity of *B. cinerea* and *P. digitatum* was evaluated by observing the uptake of propidium iodide (Sigma Aldrich, USA). The spore solutions were mixed with distilled water (untreated) or coating solution followed by shaking-incubation for 4 h, at 100 rpm, and at room temperature. Then, the samples were stained with 50 (mg L^−1^) propidium iodide and examined using a CLSM with excitation and emission wavelengths of 543 nm and 585 nm. The dead cells were indicated by propidium iodide fluorescent staining.

6 $$Percentage\,\, of\,\, staining\, \% =\frac{Stained\,\, spores}{Total\,\, spores }\times 100\%$$

where the total spores of each observation were around 150–200 spores. The result was analyzed from triplicate observations.

### Color properties

The film specimens were prepared on a standard white plate, followed by a color reader (Konica Minolta CR-20, Japan) measurement on five different spots per sample. The CIE L*, a*, b* color method was used to measure L* (lightness), a* (green to red), and b* (blue to yellow) values of the coating films. These values were further processed to obtained ∆E* (color difference) value.

7 $$\Delta E^{*} =\sqrt{{({L}_{0}^{*}-L^{*})}^{2}+{\left({a}_{0}^{*}-a^{*}\right)}^{2}+ ({{b}_{0}^{*}-b^{*})}^{2}}$$

where *L*_0_, *a*_0_, and *b*_0_ come from a standard white plate, and the L*, a*, and b* come from the values of the films.

### Light transmission and opacity

Each thin film was firstly shaped into a rectangle. It was further attached on the cell side of a cuvette and the light transmission was read using a UV–Vis spectrophotometer (Jasco, V-530, Japan) at wavelength ranges of 200–800 nm. The opacity was determined using the following equation:

8 $$Opacity = \frac{ (A600)}{x}$$

where *A600* is the absorbance at 600 nm and *x* is the film thickness (mm). Triplicate measurements were performed for each film specimen.

### Mechanical properties

The coating films were cut into a 1 × 5 cm rectangle, and the thickness of the film was measured at five different positions. The coating films were further attached into grip pairs of the motorized force test stand (Shimpo, FGS-50E-L) equipped with digital force gauge (Shimpo FGPX-50). The initial gap separation was set to 30 mm, then stretched by moving the grip with a speed of 60 mm/s until breaking. The triplicate measurements were performed. Values for tensile strength and elongation were calculated using the following equations:

9 $$Tensile\,\, strength\, ({MPa}) =\frac{ Fmax}{A}$$

where *Fmax* represents the max load (N) used to pull the film and *A* is the cross sectional area (m^2^) of the film.

10 $$Elongation\, \% =\frac{Imax}{Io }\times 100$$

where *Imax* represents the film elongation (mm) at that moment of rupture and *Io* is the original grip length (mm) of the film.

### Scanning electron microscopy

Thin coating films were pre-conditioned in a desiccator containing saturated salt of Mg_2_(NO_3_)_2_, at 18 ± 2 °C. The film specimens were placed on a specimen stub and coated in vacuum conditions with the aid of an osmium coater. Then, cross-section images were captured using a scanning electron microscope (SU3500, Hitachi, Japan) at 15 kV.

### Statistical analysis

The experimental data were analyzed through analysis of variance (ANOVA) at a significance level of *P* value < 0.05. Post hoc testing was performed using Duncan's multiple range test (DMRT) with the aid of Statistical Package for Social Science software (SPSS 17.0, SPSS Inc., USA).

## Supplementary Information

Supplementary Information.

## Data Availability

The data that support the findings of this study are available from the corresponding author upon reasonable request.
