# Supplementary material for: Antifungal features and properties of chitosan/sandalwood oil Pickering emulsion coating stabilized by appropriate cellulose nanofiber dosage for fresh fruit application
Source: Sci Rep. 2021 Sep 16;11:18412. doi: 10.1038/s41598-021-98074-w (PMC8445958; doi:10.1038/s41598-021-98074-w)
Supplement: Supplementary file 1 — Supplementary Information. [file 41598_2021_98074_MOESM1_ESM.docx]

FTIR analysis

The FTIR spectrometer (Jasco, FTIR-620, Japan) was used to analyze the chemical interaction of each coating material. The CNF, SEO, and CS were analyzed using potassium bromide pellet method at wave numbers of 375–4000 cm^-1^. The thin coating films were analyzed using an attenuated total reflectance (ATR) method at wave numbers of 500–3500 cm^-1^.

FTIR is well known as an important method for confirming structural information^1^. Figure 1 shows the FTIR spectra for the samples in the range 500–3750 cm^−1^. The presence of an absorption peak at 1652 cm^−1^ was due to the N–H bending vibration of protonated amino (−NH_2_) group and C–H bending vibration of the alkyl group. The absorption peaks at 1083 and 895 cm^−1^ were recognized due to the -symmetric stretching vibration of the C–O–C bridges and assigned to the glucopyranose ring in in the CS matrix. The band at 1595 cm^−1^ for CNFs obtained is associated with adsorbed water in cellulose. The peaks observed around 1419 cm^−1^ were attributed to the symmetric bending of CH_2_ and also related to cellulose I, the band at 1323–1373 cm^−1^ corresponded to the bending vibrations of the C–H and C–O groups of polysaccharides. The absorbance peaks observed in the 1159 cm^−1^ range were attributed to C–O–C asymmetric stretching vibrations associated with cellulose I and cellulose II. The obtained spectra around 900 cm^−1^, which was attributed to interactions between glycosidic linkages and glucose units of cellulose. FTIR spectra of SEO exhibited a characteristic absorption peak, which was in agreement with earlier studies^2,3,4^. The presence of absorption band for peaks at 1375 cm^-1^ and 1542 cm^-1^ were attributed to the methyl, methylene, and C-H stretching from SEO terpenes. The peaks at 1642 cm^-1^ and 2930 cm^-1^ were due to vibration of the aromatic ring C=C skeleton (aromatic substance) and C–H stretching band of fatty acids, respectively.


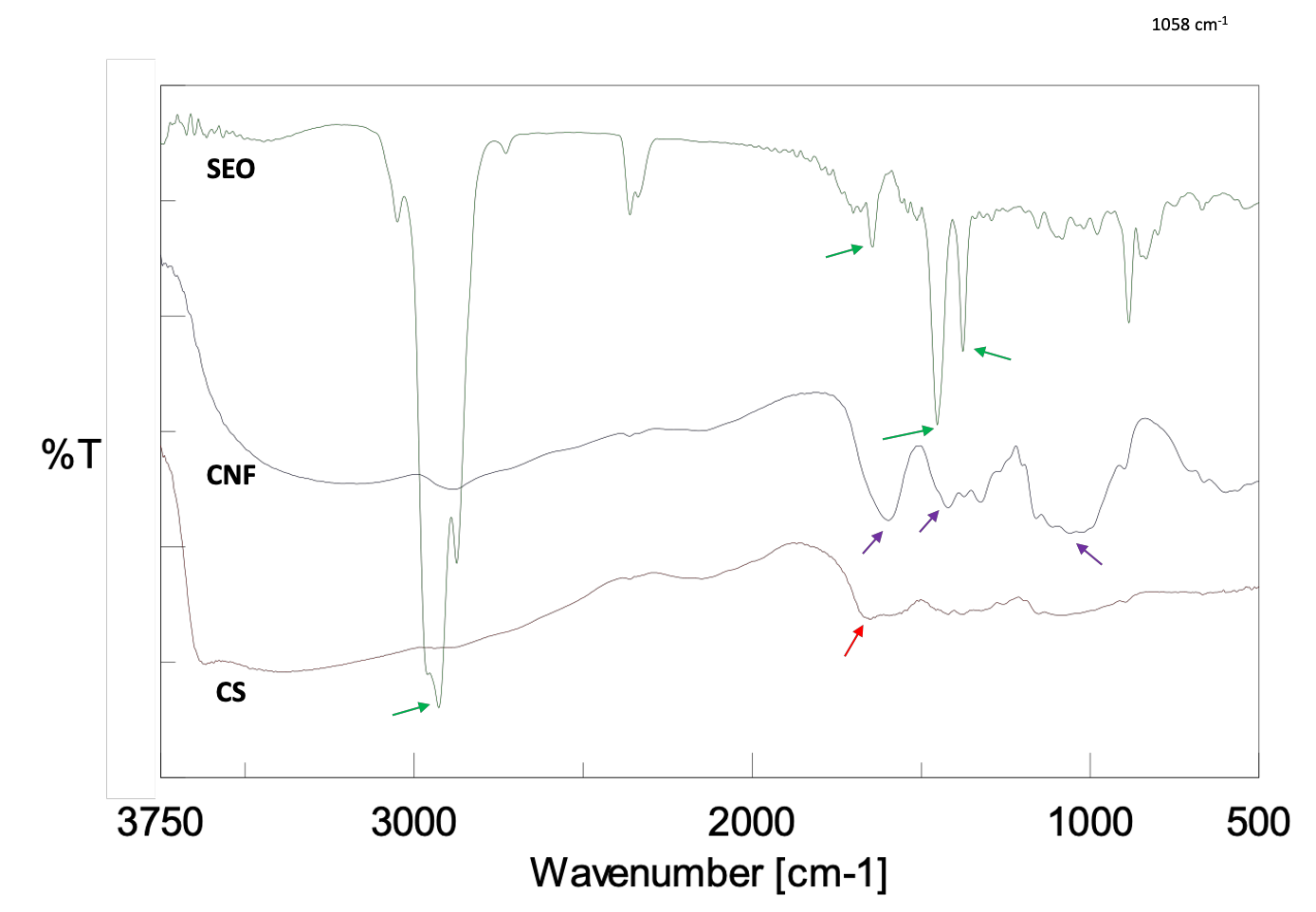


Figure 1. FTIR spectra for SEO, CNF, and CS.

In order to confirm the interactions between each composite film component, a thin film was characterized using FTIR. The FTIR spectra of emulsified films were compared with the FTIR spectrum of CS (Figure 2). The bands at 3200–3400 cm^-1^, 1638 cm^-1^, 1549 cm^-1^, 1404 cm^-1^, and 1019 cm^-1^ in the CH sample represent stretching vibrations of O–H and N–H bonds, N–H bending (amide II), C=O stretching (amide I), stretching vibrations of C–N, and stretching vibrations of C–O–C, respectively. Wavenumbers between 3200 and 3400 cm^−1^ of the emulsified films showed stronger intensity than CS alone, suggesting enhancement of the hydrogen bonding interactions between components and hydroxyl groups in the film from CNFs and SEO. In addition, the characteristic band at 2878 cm^−1^ m was higher than CS alone, indicating an increasing number of all hydrocarbon (CH) constituents in the CH-SEO and CH-SEOpick films. The absence of more prominent typical bands for SEO and CNF was potentially ascribed to the overlap of these bands with typical bands of CS and might correspond to their amount.


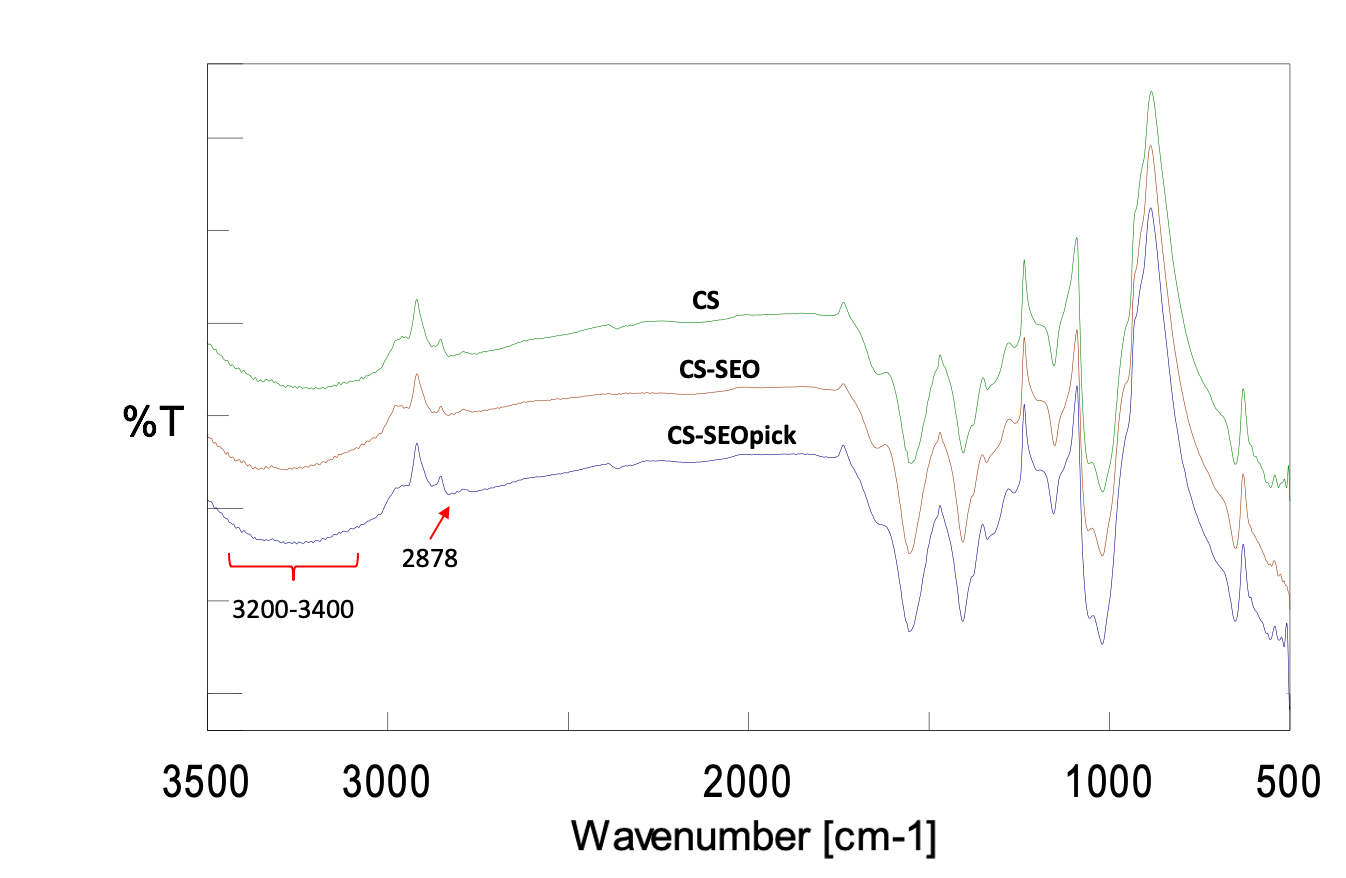


Figure 2. FTIR spectra of thin coating films.

References

1. Alharbi, N. D. & Guirguis, O. W. Macrostructure and optical studies of hydroxypropyl cellulose in pure and Nano-composites forms. *Results Phys.* **15**, 102637 (2019).

2. Baker, M. J. *et al.* Using Fourier transform IR spectroscopy to analyze biological materials. *Nat. Protoc.* **9**, 1771–1791 (2014).

3. Worzakowska, M. TG/DSC/FTIR/QMS studies on the oxidative decomposition of terpene acrylate homopolymers. *J. Therm. Anal. Calorim.* **127**, 2025–2035 (2017).

4. Kala, S., Sogan, N., Naik, S. N., Agarwal, A. & Kumar, J. Impregnation of pectin-cedarwood essential oil nanocapsules onto mini cotton bag improves larvicidal performances. *Sci. Rep.* **10**, 1–12 (2020).
